# Engineering a Dual-Function Starch–Cellulose Composite for Colon-Targeted Probiotic Delivery and Synergistic Gut Microbiota Regulation in Type 2 Diabetes Therapeutics

**DOI:** 10.3390/pharmaceutics17050663

**Published:** 2025-05-17

**Authors:** Ruixiang Liu, Yikang Ding, Yujing Xu, Qifeng Wu, Yanan Chen, Guiming Yan, Dengke Yin, Ye Yang

**Affiliations:** 1 School of Pharmacy, Anhui University of Chinese Medicine, Hefei 230012, China; lrx18226904867@163.com (R.L.); 18855221642@163.com (Y.D.); 13865247851@163.com (Y.X.); chenyananxc@163.com (Y.C.);; 2 Anhui Provincial Key Laboratory of Pharmaceutical Preparation Technology and Application, Hefei 230012, China; 3 School of Nursing, Anhui University of Chinese Medicine, Hefei 230012, China; 18056397266@163.com (Q.W.);

**Keywords:** colon-targeted drug delivery, probiotic encapsulation, starch–cellulose composite, microbiota–metabolic axis, pharmaceutical material engineering

## Abstract

**Objectives:** This study engineered a colon-targeted drug delivery system (CTDS) using the dual pharmaceutical and edible properties of *Pueraria lobata* to encapsulate *Lactobacillus paracasei* for Type 2 diabetes mellitus (T2DM) therapy. **Methods:** The CTDS was designed as a core–shell composite through microwave–hydrothermal engineering, comprising the following: (1) a retrograded starch shell with acid/enzyme-resistant crystallinity to protect probiotics from gastric degradation; (2) a porous cellulose core derived from *Pueraria lobata*’s natural microstructure, serving as a colonization scaffold for probiotics. **Results:** Structural characterization confirmed the shell’s resistance to acidic/pancreatic conditions and the core’s hierarchical porosity for bacterial encapsulation. pH/enzyme-responsive release kinetics were validated via fluorescence imaging, demonstrating targeted probiotic delivery to the colon with minimal gastric leakage. In diabetic models, the CTDS significantly reduced fasting blood glucose and improved dyslipidemia, while histopathological analysis revealed restored hepatic and pancreatic tissue architecture. Pharmacologically, the system acted as both a probiotic delivery vehicle and a microbiota modulator, selectively enriching *Allobaculum* and other short-chain fatty acid (SCFA)-producing bacteria to enhance SCFA biosynthesis and metabolic homeostasis. The CTDS further exhibited direct compression compatibility, enabling its translation into scalable oral dosage forms (e.g., tablets). **Conclusions:** By integrating natural material engineering, microbiota-targeted delivery, and tissue repair, this platform bridges the gap between pharmaceutical-grade probiotic protection and metabolic intervention in T2DM.

## 1. Introduction

Type 2 diabetes mellitus (T2DM) is a metabolic disorder characterized by insulin resistance and relative insulin deficiency [1]. Over the past decades, T2DM has been associated with significant complications, such as cardiovascular disease, nephropathy, and neuropathy, which reduce functional capacity and increase healthcare dependence [2]. Its growing prevalence places a significant burden on global healthcare systems and highlights the urgent need for effective disease management strategies.

Gut microbiota play a vital role in regulating metabolic [3], immune [4], and neuroendocrine functions [5]. T2DM is often accompanied by reduced microbial diversity and altered gut community composition. This dysbiosis involves decreased levels of beneficial bacteria, such as *Faecalibacterium*, *Roseburia*, *Bifidobacterium*, and *Lactobacillus* [6], alongside increases in pro-inflammatory taxa, such as *Clostridia* and *Escherichia*, and an increased abundance of harmful gut microbiota, particularly from the *Enterobacteriaceae* family [7]. Diet plays a crucial role in gut microbiota regulation, particularly through dietary fiber [8]. Dietary fiber escapes host enzymatic digestion and absorption in the upper gastrointestinal tract, undergoing microbial fermentation predominantly in the colon mediated by gut microbiota [9]. The metabolites derived from microbial activity provide energy to the host and contribute to the modulation of host immune function [10]. Consequently, clinical guidelines recommend dietary fiber supplementation for T2DM management [11]. Additionally, oral probiotic delivery represents another strategy to alleviate gut microbiota dysbiosis [12]. Certain probiotics help regulate fasting blood glucose (FBG) levels [13], mitigate diabetes-related tissue damage [14], and provide a robust defense against diabetes complications [15]. Despite these benefits, many probiotic strains must maintain a viability of at least 10^6^ CFU/g to be effective [16]. The highly acidic environment of the stomach presents a major challenge to probiotic survival. Therefore, an appropriate delivery system is crucial for ensuring effective oral probiotic administration.

Starch has emerged as an ideal biopolymer for probiotic encapsulation due to its natural digestibility, structural plasticity, and intrinsic prebiotic properties. Unlike synthetic polymers requiring chemical crosslinkers (e.g., glutaraldehyde), starch can be functionally modified through purely physical methods, ensuring food-grade safety and regulatory compliance [17]. Microwave–hydrothermal and cooling processing exemplify this advantage, employing sequential gelatinization–retrogradation to engineer hierarchical architectures. During microwave gelatinization, rapid dielectric heating induces starch granule swelling [18], and subsequent cooling drives amylose retrogradation, where linear chains reorganize into type III resistant starch crystals through inter-chain hydrogen bonding [19]. These retrograded starch crystals form continuous porous matrices with a significantly enhanced resistance to pancreatic α-amylase hydrolysis compared to unmodified starch [20]. Unlike enzymatic hydrolysis, which reduces the fermentability of starch, or chemical esterification, which introduces toxic residues, this method achieves dual functionality: the effective encapsulation of probiotics and the preservation of the prebiotic properties inherent to starch.

*Pueraria lobata* (the dried root of *Pueraria lobata* (Willd.) *Ohwi*) contains a significant amount of starch [21] and is widely used as a dietary supplement [22]. The microwave–hydrothermal and cooling processing of *Pueraria lobata* powder capitalizes on its dual botanical advantages: an exceptionally high starch content and evolutionarily optimized cellular architecture [23]. Microwave irradiation induces dielectric heating through polar water molecule oscillation, generating rapid starch gelatinization and volumetric expansion that mechanically disrupts parenchymal cell walls, thereby releasing starch components while preserving the microporous cellulose skeleton [24]. Cooling induces amylose retrogradation, forming anti-digestive double helices via hydrogen bonding [25]. These crystalline domains physically interlock with cellulose fibrils, establishing a core–shell composite: a porous cellulose core serving as a probiotic reservoir, encapsulated within a continuous retrograded starch shell resistant to gastrointestinal enzymes. This architecture confers pH/enzyme-responsive release kinetics, with gastric and intestinal integrity maintained via crystalline shell resistance [26,27], followed by the colonic microbiota-mediated degradation of the starch matrix to synchronize probiotic liberation and puerarin release. Notably, the mild thermal processing preserves the bioactivity of the puerarin, enabling the concurrent delivery of this β-cell-protective isoflavone alongside microbiota-modulating components [28], thereby amplifying the intrinsic anti-diabetic efficacy associated with *Pueraria lobata* through dual metabolic and regenerative pathways.

This study develops a plant-derived colon-targeting delivery system (CTDS) based on *Pueraria lobata*, engineered to synergistically enhance probiotic viability and anti-diabetic efficacy through a hierarchical architecture. Our hypothesis posits that continuous microwave–hydrothermal processing reconfigures the native starch–cellulose matrix of *Pueraria lobata* into a core–shell architecture, enabling simultaneous probiotic protection and puerarin delivery. By exploiting the dual physiological role of the colon as the primary site for probiotic colonization and prebiotic metabolism [29], this system maximizes localized therapeutic effects and minimizes systemic dispersion. Current delivery strategies face critical limitations: synthetic polymer coatings, while offering precise release control through pH-responsive mechanisms, require toxic crosslinkers that compromise biocompatibility, presenting challenges for long-term therapeutic applications [30]; natural polysaccharide matrices, exemplified by alginate–chitosan, exhibit gastric instability and always lead to premature probiotic release [31]; and conventional starch carriers from corn or potato, though widely available, lack structural robustness due to their reliance on isolated starch components [32,33]. To address these limitations comprehensively, this CTDS integrates three intrinsic properties of *Pueraria lobata*: The starch of *Pueraria lobata* (amylose fraction: 18.2%) was identified as C-type starch with a crystallinity degree of 37.76% [34]. This structural configuration demonstrates enhanced resistance to enzymatic hydrolysis relative to conventional starch matrices. This property directly resolves the structural fragility of traditional starch carriers. Concurrently, the inherent cellulose–starch compartmentalization in *Pueraria lobata* eliminates dependency on synthetic crosslinkers, thereby circumventing the toxicity risks associated with polymer coatings [35]. Furthermore, the retrograded starch shell formed through thermal processing exhibits robust resistance to gastric degradation, overcoming the instability of natural polysaccharide matrices under acidic conditions. This work establishes a microwave–hydrothermal strategy for designing natural polymer-based oral formulations that integrate probiotic protection, colon-targeted delivery, and microbiota regulation, providing a chemical-free platform to address metabolic disorders through coordinated microbial and metabolic modulation.

## 2. Materials and Methods

### 2.1. Materials

*Pueraria lobata* was obtained from Anhui Kangmei Pharmaceutical Co., Ltd. (Bozhou, China), and *Lactobacillus paracasei* (*L.p.*). was sourced from the China Center Type Culture Collection (CCTCC AB 2019261). MRS Broth and MRS Medium were obtained from Microbial culture medium Qingdao Haibo (Qingdao, China). Detection kits for total cholesterol (TC, TC1217) and triglyceride (TG, TG1247) were provided by Beijing Leagene Bioengineering Institute (Beijing, China). Detection kits for high-density lipoprotein cholesterol (HDL-C, JL-T0855) and low-density lipoprotein cholesterol (LDL-C, JL-T0807) were supplied by Shanghai Jianglai Bioengineering Institute (Beijing, China). Plasmid pNZ8148-mCherry was provided by Honor Gene (Changsha, China). Streptozotocin (≥98% purity, Sigma-Aldrich, St. Louis, MO, USA, Cat# S0130) was dissolved in citrate buffer (0.1 M, pH 4.5) and used immediately for intraperitoneal injection.

### 2.2. Construction and Characterization of Pueraria Lobata-Based Carrier for CTDS

To develop a *Pueraria lobata*-based carrier for colon targeting, sequential microwave–hydrothermal and cooling treatments were conducted and a porous structure coated with crystalline starch was created with the *Pueraria lobata* particles [36]. Briefly, *Pueraria lobata* was powdered, sieved by 100-mesh sieves, mixed with water, microwaved at 210 W for 5 min (1 min microwave/1 min interval cycle), then cooled and freeze-dried at −60 °C. In the present study, a series of powdered *Pueraia lobata* samples (microwave *Pueraia lobata*, MPLs) were obtained from *Pueraria lobata*/water mixtures with weight ratios of 7:3 (MPL_70_), 6:4 (MPL_60_), 5:5 (MPL_50_), 4:6 (MPL_40_), and 3:7 (MPL_30_) to form the CTDS.

The morphological structure of the CTDS-carrier hydrothermal–cooling-processed MPLs was analyzed using scanning electron microscopy (SEM, TESCAN MIRA LMS, TESCAN, Brno-Kohoutovice, Czech Republic). The particle size distribution was measured using a laser diffraction particle size analyzer (BT-9300H Laser Particle Sizer, Bettersize, Shenyang, China). Chemical structure was analyzed using a Fourier transform infrared spectrometer (FTIR, Nicolet 7000, ThermoFisher, Bremen, Germany) with scanning range of 4000–500 cm^−1^. The thermal properties were analyzed using a differential scanning calorimeter (DSC, Basis Unit DSC 214, Netzsch, Selb, Germany) at 10 °C/min over a temperature range of 20–250 °C. The porosity was determined using mercury intrusion porosimetry with a Mercury Porosimeter (AutoPore IV 9500, Micromeritics, Norcross, GA, USA). Untreated *Pueraria lobata* powder without microwave–hydrothermal and cooling treatments was designated as PL and also analyzed for the aforementioned physicochemical characteristics.

### 2.3. Construction and Characterization of Pueraria Lobata-Based CTDS for Probiotics (CDTS@probiotics)

*L.p.* was cultured at 37 °C with continuous agitation until it reached a concentration of 1 × 10^8^ CFU/mL. Then, the probiotic culture (1 × 10^8^ CFU/mL) was mixed with different concentrations of MPL or PL powders. After filtration, probiotics absorbed onto MPL or PL powders were freeze-dried to form a stable powder. These powders served as probiotic delivery systems and were designated as MPL_70_@*L.p.*, MPL_60_@*L.p.*, MPL_50_@*L.p.*, MPL_40_@*L.p.*, MPL_30_@*L.p.*, and PL@*L.p.* The images of these samples were captured using SEM to examine the morphology and structure of the CDTSs@*L.p*. The loaded capacity of *L.p.* was quantified as described in the literature, with slight modifications [37]. Briefly, the CDTSs@*L.p.* were suspended in a 0.9% NaCl solution and incubated with shaking at 100 rpm and 37 °C for a duration of 480 min. Following this incubation period, 1 mL of the medium was collected to assess the number of viable cells. A 10-fold serial dilution of the collected sample was performed, followed by plating on MRS agar to enumerate the viable probiotic cells. The number of CFU per unit mass of *L.p.*-loading samples (CFU/g) was calculated to reflect the loading capacity.

### 2.4. Resistance to Simulated Gastric Fluid and Probiotics Release in Simulated Intestinal Fluid

Simulated gastric fluid (SGF) was prepared by dissolving 0.2% (*w*/*v*) NaCl and 0.32% (*w*/*v*) pepsin (from porcine gastric mucosa) in deionized water, followed by adjustment to pH 1.2 with HCl. Simulated intestinal fluid (SIF) was prepared by dissolving 0.68% (*w*/*v*) KH_2_PO_4_ in deionized water, adjusting to pH 6.8 with NaOH, and adding 1% (*w*/*v*) pancreatin and 0.15% (*w*/*v*) bile salts. Both solutions were filter-sterilized (0.22 μm) prior to use. The specific method for assessing the in vitro gastrointestinal (GI) stability of *L.p.* loading samples was slightly derived from the literature [38]. The samples were incubated in SGF at 37 °C with shaking at 100 rpm for 2 h. At specific time points, samples were filtered, and the residue carrying capacity of *L.p.* was determined using the gradient dilution method above. To assess the release behavior of *L.p.*, samples were incubated in SIF at 37 °C with shaking at 100 rpm for 2 h. At specific time points, 1 mL of supernatant was collected and analyzed via the gradient dilution method.

### 2.5. In Vivo Probiotics Distribution and Colonization

To investigate the distribution and colonization of probiotics in GI conditions, *L.p*. was transfected with the plasmid pNZ8148-mCherry, and the *Pueraria lobata*-based CTDS of MPL_50_@mCherry-*L.p.* was prepared by mixing the mCherry-transfected *L.p.* culture (1 × 10^8^ CFU/mL) with MPL_50_ powder in a 5:5 (*w*/*w*) ratio, followed by vacuum filtration and lyophilization to obtain a stable probiotic-loaded powder. After a single-dose gavage of MPL_50_@mCherry-*L.p.* (1 g/kg), the Kunming mice (KM, male, 6 weeks old) were anesthetized by isoflurane at specific time points and euthanized for GI tract isolation. The distribution of mCherry-*L.p.* in GI was examined using a live small-animal optical 3D imaging system, IVIS (IVIS Spectrum, PerkinElmer, Waltham, MA, USA), at an excitation wavelength of 587 nm. To assess colonization, the mice were gavaged daily, and the imaging of the GI tract was conducted 2 h after the 5th administration [38]. Additionally, a control group was included in both the distribution and colonization experiments, which received single or multiple doses of mCherry-*L.p*. suspension (equivalent probiotic amount to MPL_50_@mCherry-*L.p.*).

### 2.6. Effects of Probiotic-Loading CTDS on T2DM Model Rats

Sprague Dawley rats (SD, male, 8 weeks old) were purchased from Anhui Qingyuan Animal Co., Ltd., Hefei, China, and housed in a controlled environment at a temperature of 24 ± 2 °C and relative humidity of 55 ± 10% with a 12 h light/12 h dark cycle and free access to standard chow and water, which complied with the nutritional components of GB14924.3–2010 (China) [39] compound feed for laboratory animals. After one week of acclimation, diabetes was induced by the intraperitoneal injection of Streptozotocin at 50 mg/kg of body weight. The successful induction of diabetes was confirmed 72 h later by measuring FBG after 6 h of fasting, with glucose levels ≥ 11.1 mmol/L [40]. The T2DM model rats were randomly divided into five groups (*n* = 5 for each group) and intervened with through various means via gavage. The rats in the CDTS@probiotics intervention group received 250 mg/kg of MPL_50_@*L.p*. powder (group MPL_50_@*L.p*.); the model rats gavaged with an equivalent quantity of probiotics (*L.p*. suspension) and hydrothermal–cooling-processed *Pueraria lobata* (MPL_50_ powder) were set as group *L.p.* and group MPL_50_; The rats in the positive control (PC) group received metformin at 250 mg/kg. Additionally, the rats in the model control (MC) and normal control (NC) groups were gavaged with distilled water. All interventions lasted 14 days and were performed once daily.

Body weight was recorded daily, and FBG was measured every three days. On the 13th day of the various interventions, fresh feces from each rat were collected under aseptic conditions for the analysis of microbial diversity and SCFAs. These analyses were performed by Suzhou Panomix Biotechnology Co., Ltd. (Suzhou, China). The microbiota diversity was analyzed using 16S rDNA sequencing, with standard bioinformatics analysis conducted on the Illumina platform. The resulting data were reanalyzed and visualized using the Bioincloud platform. SCFAs were quantified and analyzed using gas chromatography. An oral glucose tolerance test (OGTT) was conducted on the last day after a 12 h fasting period. After the treatment period, all the rats were anesthetized with isoflurane. Blood was collected from the orbital venous plexus for the serum analysis of the lipid-related parameters TC, TG, HDL-C, and LDL-C. Following blood sampling, all the rats were anesthetized with isoflurane and subsequently euthanized by cervical dislocation. Tissues from the liver, pancreas, and ileum were then collected and stained with hematoxylin & eosin (HE).

### 2.7. Storage and Heat Stability

For storage stability, the powders were, respectively, stored at −20, 4, and 20 °C for 4 weeks, and the bacterial viability was measured at regular intervals using the method mentioned above. The thermal stability of the powder was evaluated by exposing the MPL_50_@*L.p.* powder to 70, 80, and 90 °C for 20 min, and the bacterial viability was assessed. Lyophilized *L.p.* powder stored or heat-treated in the same conditions served as the control.

### 2.8. Tablet Preparation and Probiotic Release Profile

The MPL_50_@*L.p.* powder was directly compressed into tablets using a single-punch tablet press (TDP-120E, Tianhe Machinery, Shijiazhuang, China) without any additional excipient. Tablet hardness was measured using a hardness tester (PHT-6000, Phase II, Orange, CA, USA). Disintegration time was determined in distilled water at 37 ± 0.5 °C according to Chinese Pharmacopeia standards. The appearance of the tablets was captured using a smartphone camera before the disintegration tests.

To evaluate the viability of the probiotics under simulated GI conditions, the tablets were incubated in SGF at 37 °C for 2 h, followed by incubation in SIF at 37 °C for 4 h. Viable bacteria were quantified at specific intervals using serial dilution and plating. Lyophilized *L.p.* powder was tested under the same conditions for comparison.

### 2.9. Experimental Animal Ethics

All the animal studies were conducted in compliance with the Guidelines for the Care and Use of Laboratory Animals published by the U.S. National Institutes of Health (NIH Publication 85-23, 1996) Additionally, all animal experimental procedures were approved by the Animal Ethics Committee of Anhui University of Chinese Medicine, approval number: AHUCM-rats-2024041 (17 June 2024), AHUCM-mouse-2024206 (24 December 2024).

### 2.10. Data Analysis

All the experiments were conducted independently in triplicate, and the results were presented as the means and standard deviations. One-way analysis of variance (ANOVA) was conducted, followed by Duncan’s multiple range test, to assess the distinctions among the groups. A significance level of *p* < 0.05 was employed as the threshold for statistical significance. All statistical analyses were carried out using SPSS Statistics 27.0 (IBM Corp., Armonk, NY, USA).

## 3. Results and Discussion

### 3.1. Microwave–Hydrothermal and Cooling Processing Caused Physical Property Changes in Pueraria Lobata Powder Through Starch Gelatinization and Retrogradation

**Figure 1:**
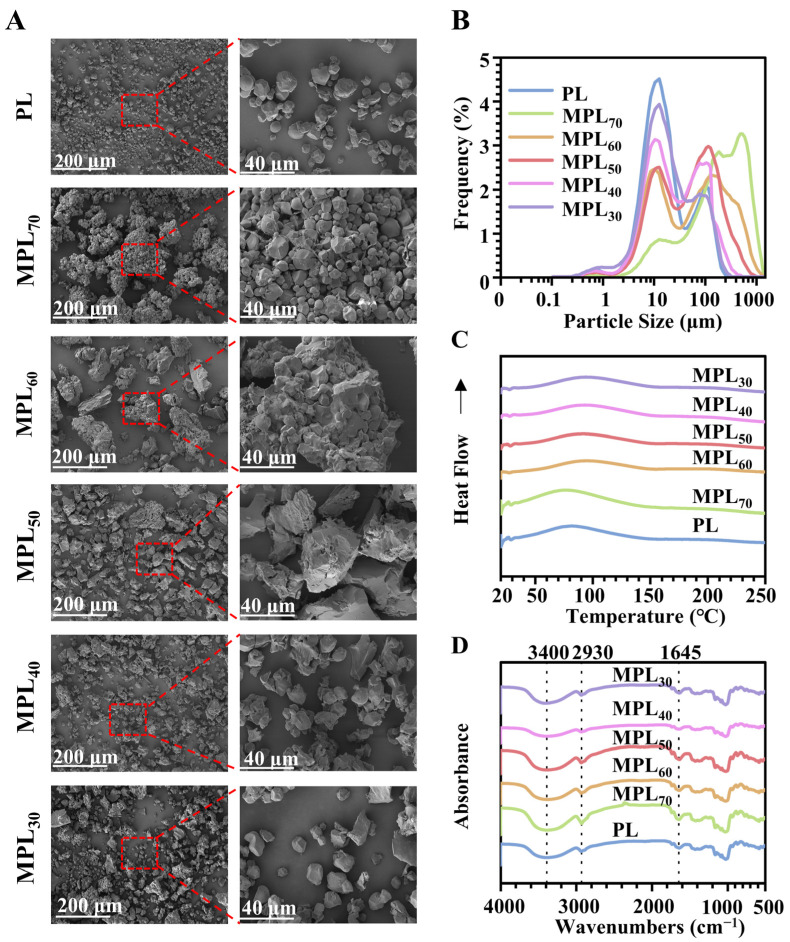
Characterizations of PL (*Pueraria lobata* powder without any treatment) and MPLs (*Pueraria lobata* powders subjected to hydrothermal–cooling processing), prepared at solid/liquid ratios of 7:3 (MPL_70_), 6:4 (MPL_60_), 5:5 (MPL_50_), 4:6 (MPL_40_), and 3:7 (MPL_30_). (**A**) SEM images; (**B**) particle size distribution; (**C**) DSC curves; (**D**) FTIR spectra.

The untreated *Pueraria lobata* powder (sample PL) was composed mainly of well-dispersed particles with uniform polyhedral or spherical shapes and smooth surfaces (Figure 1A). After hydrothermal–cooling processing, varying degrees of aggregation, volumetric expansion, pore formation, and rupture were observed. In the group with the lowest water amount during hydrothermal processing (group MPL_70_), the particles did not exhibit size and micromorphology changes but did exhibit obvious agglomeration. As the water content in the *Pueraria lobata*/water mixtures increased during microwave–hydrothermal processing (from 30% to 70%), particle agglomeration gradually decreased, while volumetric expansion, pore formation, and particle rupture progressively developed. The particle size distribution parameters of the MPL_70_ group were significantly larger than those of the PL group (*p* < 0.05), and the distribution curve exhibited a significant shift towards larger sizes (Table 1 and Figure 1B). As the water content in the *Pueraria lobata*/water mixtures increased during microwave–hydrothermal processing (from 30% to 70%), the particle size distribution parameters gradually decreased and approached those of the PL group (Table 1), as did the peak values and the shape of the size distribution curves (Figure 1B). These findings indicate that microwave–hydrothermal processing significantly modified the particle morphology and size distribution of PL and the MPLs, depending on the water content.

A higher perforation capacity for carrying materials means that they can accommodate a greater number of active probiotics. Of all the *Pueraria lobata*-based carriers, sample MPL_50_ exhibits the highest porosity and largest pore volume (Table 2). These properties might have enhanced its effectiveness as a carrier system for probiotics and helped facilitate higher bacterial survival rates in challenging environments such as the gastrointestinal tract. Table 3 summarizes the DSC parameters of the PL and MPL samples. In comparison to the PL sample, the peak temperatures (*T_p_*) of the MPL_70/60/50/40_ samples exhibited a shift towards to higher temperatures, while sample MPL_30_ exhibited a shift towards lower temperatures (Figure 1C). These thermal shifts reflect the hydrothermal-processing-induced starch chain breakage, cooling-processing-induced molecular rearrangement, and inter-chain hydrogen bond regeneration of amylose. These phenomena are attributed to the retrogradation properties of starch [41]. In the FTIR spectra, the peak at 3400 cm^−1^ is assigned to the stretching of O–H bonds and the vibrations of hydrogen bonds [21]. The peak at 2930 cm^−1^ is assigned to C–H asymmetric stretching and vibration, while the peak at 1645 cm^−1^ is assigned to O–H bending vibration in the amorphous region of the starch granules [27]. The broader peak at 3400 cm^−1^ in sample MPL_70_, compared with PL, suggests a greater number of hydrogen bonds. This phenomenon intensified with increasing water content (from 30% to 70%). These results suggest that increasing the water content during microwave processing enhances the regeneration of hydrogen bonds between amylose during cooling. This strengthened regeneration of hydrogen bonds between amylose, as shown through the DSC and FTIR analysis, may lead to unique digestion properties and enhanced probiotic delivery capacity in the intestine.

The experimental results demonstrated that the water ratio critically governed the formation and physicochemical characteristics of the “starch-coated cellulose drug delivery” composite structure by modulating starch gelatinization and retrogradation behaviors. Under low-water conditions, partial starch gelatinization occurred, followed by the hydrogen-bond-mediated reorganization of amylose during cooling to form a compact, enzyme-resistant crystalline shell. This core–shell hierarchical architecture was simultaneously stabilized through physical interlocking with the cellulose matrix [42]. The resulting gelatinized/high-crystalline structure not only exhibited enhanced thermal stability and enzymatic resistance as a functional barrier, but also delayed payload release in gastrointestinal environments through its dense shell morphology. Concurrently, the inherent porosity of the internal cellulose network provided a structural foundation for drug loading [43].

Conversely, excessive water content induced the complete gelatinization of the starch into disordered amorphous structures, which failed to reconstruct effective protective shells upon cooling. The compromised hydrogen-bond networks and collapsed pore architecture further destabilized the composite system [44]. Notably, moderate water ratios achieved an optimal equilibrium between starch expansion and cellulose skeleton preservation. This balance enabled sufficient starch gelatinization to form continuous coating layers while maintaining the innate porosity of the cellulose networks, thereby maximizing both drug-loading capacity and colon-targeted delivery efficiency. These findings confirm that the precise regulation of water content during microwave–hydrothermal processing represents a viable strategy to optimize the microstructural engineering and functional customization of *Pueraria lobata*-based carriers.

### 3.2. Microwave–Hydrothermal- and Cooling-Processed Pueraria Lobata Powder Can Serve as the Carrier Material of an Oral Probiotics Delivery System

**Figure 2:**
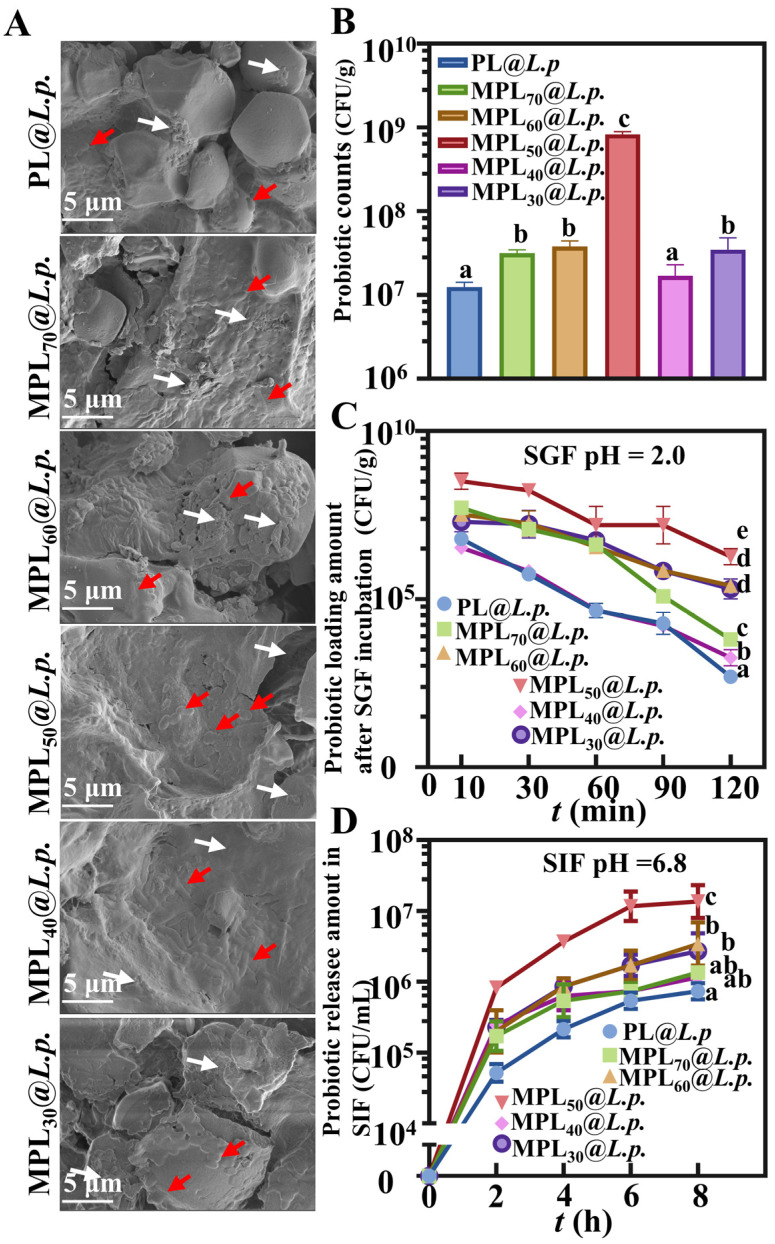
Probiotic delivery capacity of PL@*L.p*. and MPLs@*L.p.* (**A**) Representative SEM images (red arrow, intact probiotics; white arrow, broken probiotics); (**B**) probiotic loading capacity. (**C**) Resistance in SGF. (**D**) Probiotic release behavior in SIF. Significant differences indicated by different letters (*p* < 0.05); *n* = 3.

*L.p.* adhered to the surfaces of the PL and MPL samples and consistently exhibited two distinct morphologies: fully intact rods (indicated by red arrows) and fragments characterized by fragmentation, crumpling, and deformation (indicated by white arrows), as shown in Figure 2A. The MPL samples provided similar results; however, there was a noticeable disparity in the proportion of injured cells. Survival counts of *L.p.* were conducted to evaluate the loading capabilities. The *L.p.* survival counts for the MPLs@*L.p.* were always higher than those of PL@*L.p.* Among them, MPL_50_@*L.p.* had markedly greater *L.p.* survival counts than the other MPL@*L.p.* samples (Figure 2B). After 2 h of incubation in SGF, results demonstrated that MPL_50_@*L.p.* demonstrated the highest viable *L.p.* count (Figure 2C). The results of the release experiments conducted in SIF indicated that MPL_50_@*L.p.* released the highest amount of viable probiotics over an 8 h incubation (Figure 2D).

The enhanced probiotic viability and sustained release in MPL_50_@*L.p.* stem from its optimized core–shell architecture, engineered through controlled water content (50%) during microwave–hydrothermal processing. Partial starch gelatinization followed by retrogradation created a crystalline shell resistant to gastric degradation, while preserving the cellulose core’s high porosity. This structure synergistically protected probiotics via surface shielding against acid penetration and pore-mediated ice-crystal mitigation during lyophilization. Controlled intestinal release emerged from enzymatic starch degradation coupled with diffusion through the cellulose network. Suboptimal water ratios (<50%) produced incomplete crystallization, whereas excess water (>50%) caused pore collapse, highlighting water’s critical role in balancing starch–cellulose interactions. These results validate starch retrogradation [45] and scaffold-based delivery principles for plant-derived carriers [46].

### 3.3. Oral Probiotic Delivery System Could Enhance Colonization of L.p. in GI Tract

**Figure 3:**
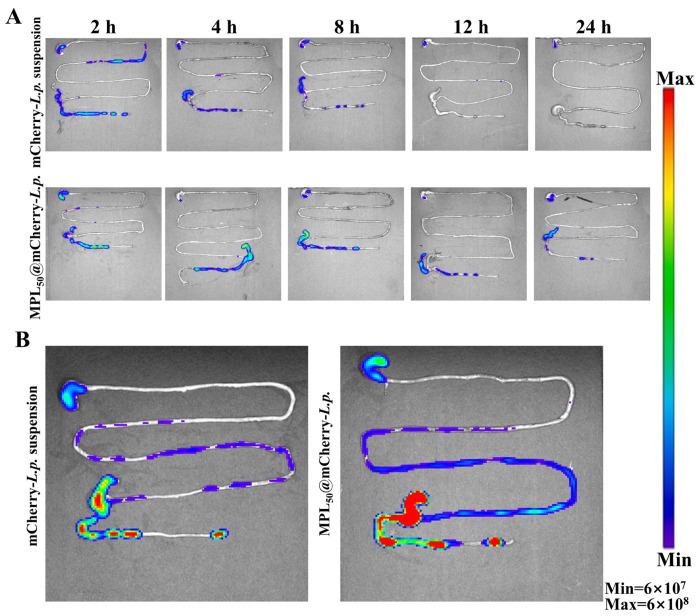
IVIS observation of probiotic retention and colonization in gastrointestinal tract after single- (**A**) or multiple-dose (**B**) gavage.

To access the survival capability of probiotics in the GI tract, *L.p.* was transfected with the pNZ8148-mCherry plasmid to enable mCherry protein expression. Following both mCherry-*L.p.* suspension and MPL_50_@mCherry-*L.p.* gavage, fluorescence signals were monitored. The fluorescence data were obtained after subtracting the background fluorescence from the control mice gavaged with water. In the mCherry-*L.p.* suspension group (Figure 3A), fluorescence signals appeared strongly in stomach, ileocecal valve, and colon at 2 and 4 h post-gavage, rapidly declined in intensity in the later stage, and disappeared entirely at 12 h post-gavage. In the MPL_50_@mCherry-*L.p.* group, fluorescence signals were strongly observed in the stomach and ileocecal regions at 2 h post-gavage, weakened in the stomach by 4 h, but persistently existed in the ileocecal regions throughout the experiment period, even when the fluorescence signals in colon had disappeared within the feces 24 h later. Fluorescence signals were persistently observed in the ileocecal junction and colon, but their intensity gradually diminished over time. To examine the colonization and proliferation of probiotics in the GI tract, multiple doses of mCherry-*L.p.* suspension and MPL_50_@mCherry-*L.p.* were gavaged to the mice. In the mCherry-*L.p.* suspension group, as shown in Figure 3B, fluorescence signals were mainly observed in the stomach, ileocecal valve, and colon, and scattered in the ileum. In the MPL_50_@mCherry-*L.p.* group, fluorescence signals existed in a wide range of the GI tract, except for the duodenum, and were strong in the ileocecal junction and colon. On the whole, the fluorescence intensity in the MPL_50_@mCherry-*L.p.* group was much stronger than that in the mCherry-*L.p.* suspension group. These results indicate that, compared to mCherry-*L.p.* suspension, the CTDS of MPL_50_@mCherry-*L.p.* exhibited significantly improved probiotic survival during transit through the upper gastrointestinal tract, and the multiple-dose fluorescence signals monitored during the gavage of MPL_50_@mCherry-*L.p.* achieved the goal of probiotic colonization and proliferation in the ileocecal valve and colon.

This CTDS overcomes the persistent trade-off between biocompatibility, structural stability, and functional precision that has constrained existing probiotic delivery platforms. While synthetic encapsulation strategies can achieve controlled release profiles through pH-responsive mechanisms, their dependence on chemical crosslinkers and organic solvents introduces biological safety risks, which fundamentally limits their clinical implementation [47]. Natural polysaccharide-based carriers, while circumventing synthetic additives, face intrinsic instability in gastric environments due to pH- or enzyme-triggered degradation, resulting in premature payload release [48]. In contrast, this CTDS leverages physical engineering to preserve the natural starch–cellulose compartmentalization inherent to *Pueraria lobata*, eliminating chemical additives while retaining enzymatic and acidic resistance. This dual capability bridges the gap between biocompatibility and functional robustness, offering a paradigm distinct from existing systems.

Static in vitro systems, though valuable for controlled parameter analysis, often inadequately replicate dynamic gastrointestinal conditions such as peristaltic forces and microbiota interactions, leading to inflated efficacy predictions [49]. Advanced co-culture models, while incorporating multiple cellular components, fail to recapitulate dynamic physiological factors like peristalsis and biochemical gradients [50]. Large mammalian models, while offering anatomical and physiological similarities to humans, are hindered by prohibitive costs, technical complexity, and persistent interspecies microbiota differences [51]. To address these challenges, our study implemented a dual-imaging approach: the ex vivo imaging of murine intestinal segments was integrated with concurrent in vivo monitoring. This strategy preserved native tissue architecture while capturing site-specific retention dynamics across physiological compartments. This experimental design strategically exploits the practical advantages of rodent models, particularly their shorter experimental timelines, to achieve a high-resolution characterization of probiotic delivery dynamics. This hybrid approach captures probiotic transit dynamics with spatiotemporal precision, bridging the mechanistic insights of reductionist models and the functional relevance of whole-animal studies.

### 3.4. Colon-Targeting Probiotic Delivery System Could Improve Oral Glucose Tolerance and Restore Dyslipidemia in T2DM Model Rats

**Figure 4:**
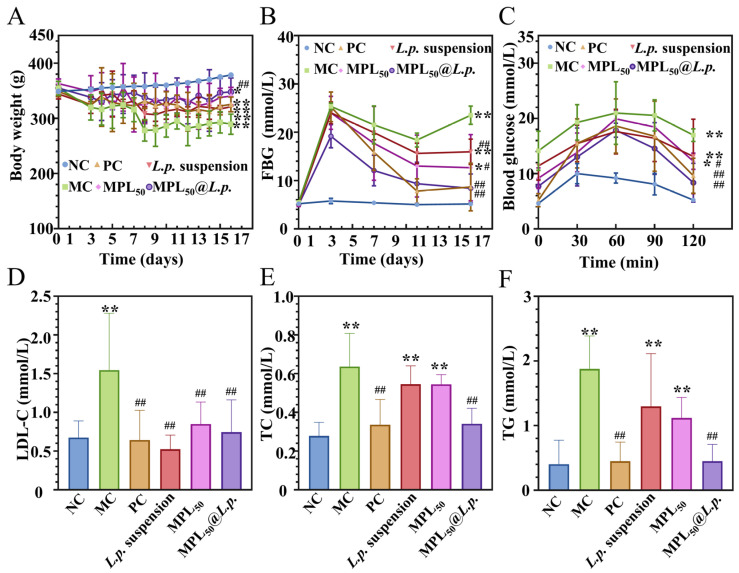
Effects of MPL_50_@*L.p.* colonic targeting probiotic delivery system on physiological and biochemical parameters of T2DM model rats. (**A**) Body weight; (**B**) FBG; (**C**) blood glucose during oral glucose tolerance test; (**D**) LDL-C; (**E**) TC; (**F**) TG. ** and *, *p* < 0.01 and 0.05 vs. NC group; ## and #, *p* < 0.01 and 0.05 vs. MC group; *n* = 3.

During the whole experiment, the rats in the NC group exhibited a gradual increase in body weight (Figure 4A) and maintained a steady FBG level (Figure 4B) within the healthy range of 4–7 mmol/L. In contrast, the rats injected with STZ exhibited the diabetic symptoms of polyuria, polydipsia, and polyphagia. The rats in the MC group showed a gradual decrease in body weight and a persistent increase in FBG. All the intervention groups in the present study could alleviate the body weight loss and restore the abnormal FBG of the diabetic rats. The OGTT results showed that the blood glucose of the normal rats (NC group) rose over 60 min and gradually returned to a healthy range (Figure 4C), and STZ induction significantly elevated blood glucose levels. Compared with the MC group, the interventions of metformin, the *L.p.* suspension, hydrothermal–cooling-processed *Pueraria lobata* powder (MPL_50_), and CTDS MPL_50_@*L.p.* induced significant (*p* < 0.05) decrease in the area under the curve of blood glucose curves. Diabetic dyslipidemia is a frequent consequence of diabetes. The rats in the MC group exhibited significantly (*p* < 0.05) higher levels of LDL-C (Figure 4D), TC (Figure 4E), and TG (Figure 4F) than the normal rats in the NC group. The *L.p*. suspension and hydrothermal–cooling-processed *Pueraria lobata* powder (MPL_50_) interventions significantly (*p* < 0.05) reduced the aberrant LDL-C level, but did not have a significant impact on TC or TG levels. Additionally, oral treatment with metformin and MPL_50_@*L.p.* reduced (*p* < 0.05) all the aberrant indices of lipid metabolism in the diabetic model rats.

Hyperglycemia may impair lipid oxidation via substrate competition for entry into cellular metabolism, hindering the utilization of lipid oxidation. Metformin can improve the metabolism of abnormal blood lipids, which may be related to the metformin-induced enhancement of glucose uptake and utilization [52]. The *L.p.* suspension and hydrothermal–cooling-processed *Pueraria lobate* powder (MPL_50_) interventions only reduced LDL-C levels, which might because of their minor effects on FBG and oral glucose tolerance. However, the CTDS of MPL_50_@*L.p.* indicated a significant promotion of both glucose and lipid metabolic disorders in the T2DM model rats.

### 3.5. Colon-Targeting Probiotic Delivery System Restored Histological Abnormality of Liver, Pancreas, and Ileum in T2DM Model Rats

**Figure 5:**
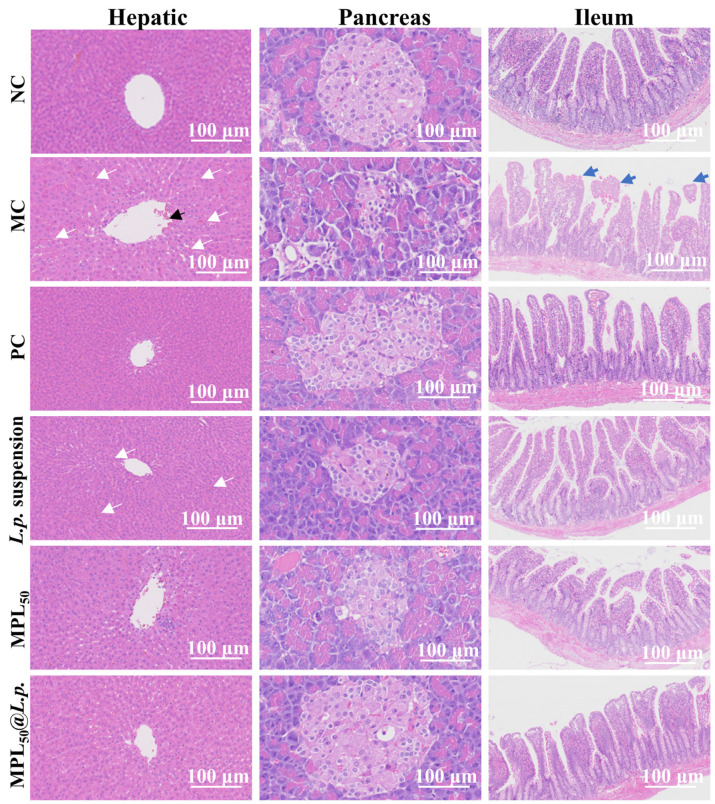
Representative images of H&E staining of hepatic, pancreatic, and ileum tissues. (Black arrows: enlarged hepatic central veins; white arrows: disordered hepatocytes and congested hepatic sinusoids; blue arrows: ileal mucosal erosion/villi rupture).

Figure 5 depicts representative histological micrographs of hepatic, pancreatic, and ileum tissues across experimental groups, with arrows highlighting key pathological or restorative features. In the NC group, the hepatic tissues exhibited well-defined lobules and neatly arranged hepatocytes with a normal size, shape, and distribution; the size and number of the pancreatic islets were normal, and the shape, size, and arrangement of the pancreatic cells were normal and regular; and the ileum tissues had conspicuous crypts and neatly distributed villi. In the MC group, the central veins in the hepatic tissues were enlarged (black arrows), the hepatocytes were disordered, and the hepatic sinusoids were congested (white arrows); the pancreatic islets were atrophic, structurally disordered with blurred peripheral boundaries, and showed cytoplasmic vacuolation; and the ileum tissues exhibited histological features of mucous membrane erosion or loss and villi rupture (blue arrows). After intervention via metformin (PC group), the central veins in the hepatic tissues were restored; the pancreatic tissue exhibited an intact pancreatic islet structure and clear boundaries; and the ileum tissue exhibited some damaged villi. After intervention via the *L.p.* suspension, the central veins in the hepatic tissue returned to their normal size, but there was a degree of congestion in adjacent hepatic sinusoids; the pancreatic tissue exhibited an intact pancreatic islet structure and clear boundaries, but the reduced islet cell number had not been recovered; and the ileum had clear crypts and evenly spaced villi. After intervention with the hydrothermal–cooling *Pueraria lobata* powder (MPL_50_), the hepatic tissue exhibited well-defined lobules and neatly arranged hepatocytes; the pancreatic tissue exhibited a clear pancreatic islet boundary but a not fully recovered islet cell number; and the ileum exhibited mucous membrane erosion and loss and villi rupture. After intervention with the colon-targeting probiotic delivery system (MPL_50_@*L.p.*), the hepatic tissue showed clear lobules and neatly arranged hepatocytes; the pancreatic islets were well-structured and the pancreatic cells remained clean and well-ordered; and the ileum tissue had conspicuous crypts and neatly distributed villi.

In the management of T2DM, addressing persistent hyperglycemia and its associated tissue damage requires multifactorial interventions. Synergistic delivery strategies provide a viable framework for such multidimensional interventions. For example, the co-administration of dietary fiber with specific *Lactobacillus* strains has been shown to amplify short-chain fatty acid production, thereby enhancing insulin sensitivity more effectively than either component alone [53]. Similarly, polyphenol-rich extracts, when combined with probiotics, demonstrate compounded anti-inflammatory effects through the dual modulation of gut barrier integrity and macrophage polarization [54]. Berberine and paeoniflorin synergistically counteract T2DM through multi-target mechanisms, effectively lowering blood glucose and insulin resistance, demonstrating the potential of herbal combination therapies [55]. Although probiotics and *Pueraria lobata* exhibit complementary mechanisms for diabetes management, their individual applications present limitations. Probiotics primarily improve glucose metabolism through the modulation of gut microbiota, whereas *Pueraria lobata* demonstrates dual functionality: the isoflavone puerarin within the root directly rejuvenates pancreatic β-cells, while the structural fibers of the root provide prebiotic substrates. To address these constraints, we leveraged the intrinsic hierarchical architecture of *Pueraria lobata* through microwave–hydrothermal processing. This method converts the native starch–cellulose matrix into a colon-targeted delivery system with dual mechanisms: retrograded starch forms a gastric-resistant protective layer for probiotics and cellulose fibers encapsulate puerarin to regulate its release kinetics. Crucially, microbial enzymes derived from colonic microbiota degrade the retrograded starch component in a controlled manner, thereby synchronizing the liberation of viable probiotics and bioactive puerarin. Compared to synthetic co-delivery systems, dependent on potentially toxic chemical crosslinkers, this plant-based platform achieves the spatiotemporal coordination of therapeutic agent delivery through enzymatic responsiveness to colonic microbiota activity [56]. By unifying structural biocompatibility with physiological responsiveness, this platform bridges functional foods and pharmaceutical efficacy, offering a clinically viable strategy for diabetes intervention.

### 3.6. Colon-Targeting Probiotic Delivery System Promoted Proliferation of Probiotics and Facilitated Production of SCFAs

**Figure 6:**
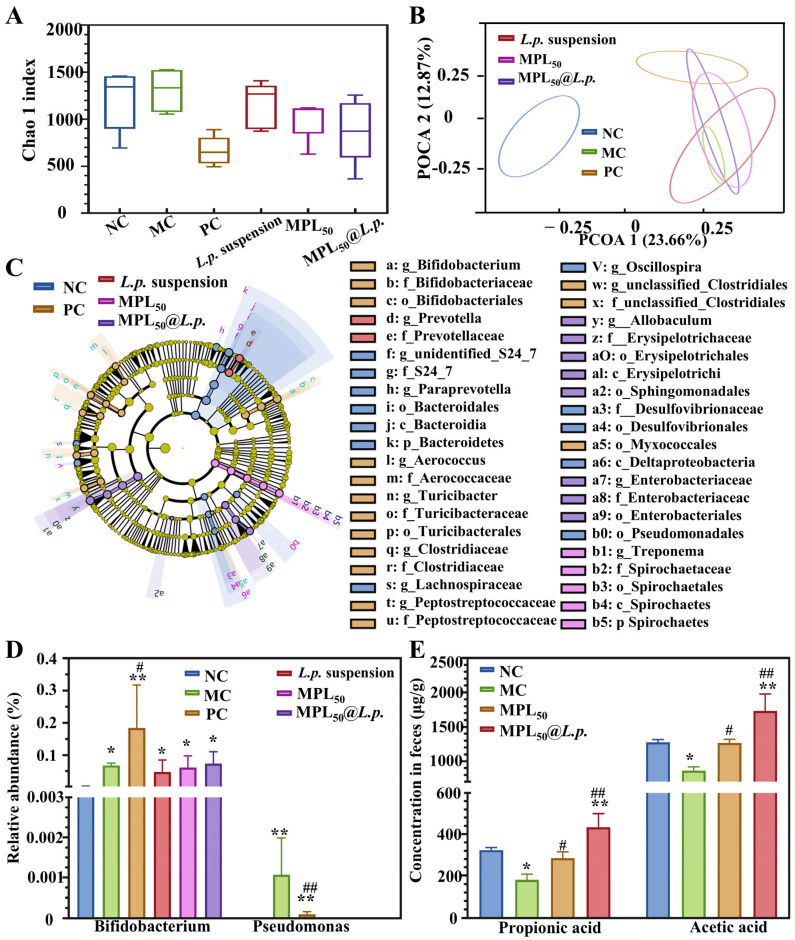
Effects of MPL_50_@*L.p.* on gut microbiota composition and SCFA levels in T2DM rats. (**A**) Chao 1 index; (**B**) Principal Coordinates Analysis; (**C**) linear discriminant analysis effect size (LEfSe), highlighting marker species at various taxonomic levels; (**D**) genus-level analysis showing quantitative differences in microbial communities between MC and PC; (**E**) quantification of SCFAs in gut, including two most abundant SCFAs, acetic acid and propionic acid, in NC, MC, MPL_50_, and MPL_50_@*L.p.* groups. Samples for flora analysis and short-chain fatty acid quantification obtained from feces. ** and *, *p* < 0.01 and 0.05 vs. NC group; ## and #, *p* < 0.01 and 0.05 vs. MC group; *n* = 5.

α-diversity and β-diversity indices reflect species diversity within a specific ecosystem and across different ecosystems, respectively, providing insights into species richness and distribution. Our analysis revealed no significant differences in α-diversity among the groups (Figure 6A). However, β-diversity analysis indicated significant differences in community composition between the MC group and the NC group. Additionally, distinct disparities were observed in community profiles when comparing the PC, *L.p.*, MPL_50_, and MPL_50_@*L.p.* groups with the MC group (Figure 6B). Further analysis through LEfSe highlighted these notable distinctions by identifying the “marker species” at various taxonomic hierarchies compared to other groups. The NC group revealed genus-level biomarkers of *Paraprevotella* and *Oscillospira* (Figure 6C). *Paraprevotella* is a known probiotic genus, often showing depletion in diabetic states in rats [57]. *Oscillospira* is helpful in improving lipid metabolism and mitigating obesity [58]. In the PC group, the biomarkers included *Bifidobacterium* and *Aerococcus*. While *Bifidobacterium* serves as a probiotic in the GI tract, *Aerococcus* might be linked to inflammatory processes [59]. The biomarker in *L.p.* suspension group was the genus *Prevotella*, which aids in protein and carbohydrate metabolism [60]. Meanwhile, the MPL_50_ group biomarkers fall within the phylum Spirochaetes and genus *Treponema*. *Treponema* contributes to SCFA synthesis. The MPL_50_@*L.p.* group was marked by *Allobaculum*, capable of producing acetic acid through non-digestible carbohydrate fermentation [61]. The model group showed a significant increase in the harmful bacterial genus *Pseudomonas*. In the PC group, *Bifidobacterium* levels significantly increased (Figure 6D).

Acetic acid and propionic acid are the most abundant SCFAs produced by intestinal microorganisms [62]. In the MC group, the levels of propionic acid and acetic acid significantly decreased (*p* < 0.05). This reduction was likely due to the decreased probiotic abundance in the intestine. However, the interventions with MPL_50_ and MPL_50_@*L.p.* reversed these reductions in SCFAs (Figure 6E). The modulation of gut microbiota by MPL_50_ and MPL_50_@*L.p.* influences the production of these vital SCFAs. Acetic acid can be used by the body as a source of energy and is involved in lipid and glucose metabolism [63]; propionic acid not only serves as a source of energy but also participates in glucose synthesis in the hepatic system and modulates hepatic gluconeogenesis to stabilize glycemia [64].

This study underscores the pivotal role of gut microbiota in metabolic regulation, particularly through the production of SCFAs. Among the interventions, MPL_50_@*L.p.* exhibited the most remarkable effects, effectively restoring levels of acetic acid and propionic acid while reshaping the gut microbial community. The identification of *Allobaculum* as a key biomarker in the MPL_50_@*L.p.* group highlights its ability to enhance non-digestible carbohydrate fermentation and acetic acid synthesis. These results suggest that MPL_50_@*L.p.* may serve as a promising approach to alleviating gut dysbiosis and supporting metabolic health.

### 3.7. MPL50@L.p. Powder Had Comparable Storage and Thermal Stabilities with Lyophilized L.p. Powder and Could Easily Be Tableted

**Figure 7:**
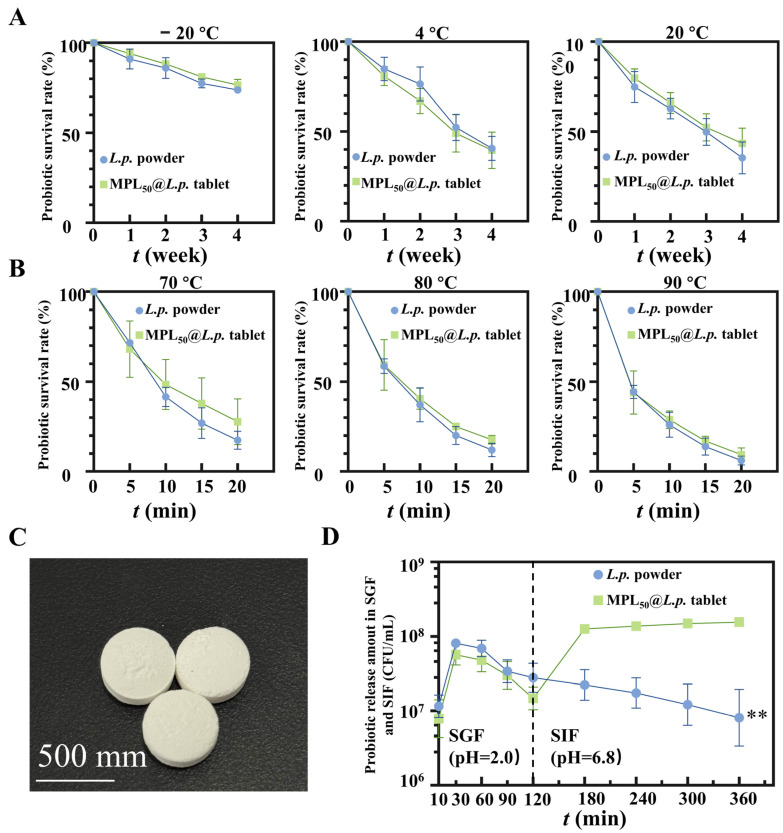
Storage and heat stabilities of MPL_50_@*L.p.* powder and probiotic release profile of MPL_50_@*L.p.* tablets. (**A**,**B**) Survival curves of probiotics in MPL_50_@*L.p.* powder after storing at −20, 4, and 20 °C (**A**) and heat-treating at 70, 80, and 90 °C (**B**). Comparison with lyophilized *L.p.* powder. (**C**) Photograph of MPL_50_@*L.p.* tablets. (**D**) Probiotic release amount of lyophilized *L.p.* powder and MPL_50_@*L.p.* tablets during SGF and SIF incubation. **, *p* < 0.01 vs. MPL_50_@*L.p.* tablet group; *n* = 3.

As shown in Figure 7A, the MPL_50_@*L.p.* powder exhibited similar storage and thermal stabilities to the *L.p.* lyophilized powder. After 4 weeks of storage at −20 °C, the viability of the bacteria in the MPL_50_@*L.p.* powder remained at 74.2 ± 3.2%. However, when stored at 4 or 20 °C, the bacterial viability decreased rapidly, and the survival rates of *L.p.* in MPL_50_@*L.p.* after 4 weeks storage dropped to 39.6 ± 10.1% and 43.5 ± 8.5%, respectively. Under high-temperature conditions (70, 80, and 90 °C), the bacterial viability in the MPL_50_@*L.p.* powder declined rapidly, and remained at survival rates of 68.1 ± 15.7%, 59.3 ± 14.1%, and 44 ± 12.0% after 5 min of exposure, respectively (Figure 7B).

To enhance the practical application of MPL_50_@*L.p.* in diabetes management, the powder was directly compressed into tablets without additional excipients. The resulting tablets were white, with a smooth and intact surface free of cracks (Figure 7C). The hardness of the tablets was 65.5 ± 5.2 N, and the in vitro disintegration time was 406 ± 29 s. These features were all met the requirements of the China Pharmacopeia (2020). During the in vitro evaluation of probiotic releasing from the MPL_50_@*L.p.* tablets (Figure 7D), the viable bacteria in the SGF after 30 min of incubation was (5.9 ± 1.6) × 10^7^ CFU/mL. This likely resulted from the disintegration of the tablet and the release of bacteria near the surface of the MPL_50_ particles. Subsequently, the number of viable bacteria in the incubation medium decreased steadily due to the acidic environment. After transitioning to SIF incubation, the bacterial count in the medium gradually increased. In contrast, when an equivalent dose of *L.p.* lyophilized powder was subjected to the same SGF and SIF incubation, the bacterial amount in the medium continuously declined. After 2 h of incubation in SGF, only 8.8 ± 2.4% of viable bacteria remained, indicating that most bacteria perished in the highly acidic environment. Briefly, MPL_50_@*L.p.* tablets provided superior protection for probiotic viability under simulated gastrointestinal conditions. The CTDS developed in this study offers a scalable and cost-effective alternative to synthetic encapsulation technologies. Its compatibility with direct-compression tablet formulations enhances its translational potential for functional food and pharmaceutical applications.

## 4. Conclusions

This study establishes a microwave–hydrothermal-processed pharmaceutical engineering approach to develop a *Pueraria lobata*-derived CTDS for probiotic encapsulation and metabolic intervention in T2DM. By precisely controlling water-mediated structural transitions during processing, we engineered starch–cellulose composites with dual pharmaceutical functionalities: a retrograded starch matrix resistant to gastric degradation and a porous cellulose network preserving probiotic viability. Structural optimization at critical water-to-solid ratios balanced starch gelatinization–retrogradation dynamics with cellulose skeleton integrity, achieving colon-specific release kinetics through pH/enzyme-responsive starch degradation and sustained probiotic liberation.

Pharmacodynamic evaluation in a T2DM murine model demonstrated that CTDS@*L.p.* administration significantly enhanced probiotic survival under gastrointestinal conditions, restored fasting blood glucose and lipid profiles, and ameliorated histopathological damage in pancreatic and hepatic tissues. This therapeutic efficacy was attributed to dual mechanisms: (1) targeted probiotic delivery facilitated by starch retrogradation, which protected *L.p.* from gastric degradation and ensured colonic liberation; (2) microbiota–metabolic modulation driven by the CTDS-mediated enrichment of the SCFA-producing *Allobaculum*, thereby enhancing intestinal SCFA biosynthesis. Furthermore, the preserved cellulose porosity allowed for the sustained release of bioactive components from *Pueraria lobata*, contributing to systemic metabolic recovery.

This work establishes a microwave–hydrothermal strategy for designing natural polymer-based oral formulations that integrate probiotic protection, colon-targeted delivery, and microbiota regulation. Future studies should focus on scaling up the processing technique for pharmaceutical production, validating long-term stability under physiological conditions, and exploring personalized microbiota-directed interventions for metabolic disorders.

## Figures and Tables

**Table 1:** Particle size distribution parameters of PL and MPL samples.

Samples	D (10)/μm	D (50)/μm	D (90)/μm	D [2,3]/μm	D [3,4]/μm
PL	5.58 ± 0.12 ^a^	15.40 ± 0.40 ^a^	102.23 ± 0.61 ^a^	9.83 ± 0.23 ^a^	34.26 ± 0.50 ^a^
MPL_70_	16.33 ± 0.96 ^c^	199.77 ± 20.34 ^c^	748.80 ± 101.38 ^d^	41.18 ± 2.24 ^c^	304.80 ± 37.02 ^d^
MPL_60_	6.55 ± 0.08 ^b^	66.93 ± 5.13 ^b^	429.30 ± 20.80 ^c^	14.22 ± 0.30 ^b^	149.10 ± 7.64 ^c^
MPL_50_	7.21 ± 0.34 ^b^	57.90 ± 8.36 ^b^	285.50 ± 71.23 ^b^	16.49 ± 1.57 ^b^	109.69 ± 21.37 ^b^
MPL_40_	5.29 ± 0.69 ^a^	18.06 ± 7.78 ^a^	125.87 ± 23.19 ^a^	9.66 ± 1.67 ^a^	44.32 ± 11.14 ^a^
MPL_30_	5.29 ± 0.17 ^a^	16.74 ± 0.22 ^a^	99.19 ± 4.21 ^a^	8.93 ± 0.25 ^a^	35.53 ± 1.21 ^a^

Note: Significant difference is indicated by different letters within the same column for the same substrate (*p* < 0.05); *n* = 3.

**Table 2:** Porosity and pore characteristics of PL and MPL samples.

Samples	Porosity/%	Average Pore Size/nm	Total Pore Volume /mL·g^−1^
PL	52.53	2998	0.7834
MPL_70_	56.68	2138	0.9634
MPL_60_	63.43	5248	1.2883
MPL_50_	69.41	6598	1.6232
MPL_40_	64.18	5214	1.2786
MPL_30_	53.00	3408	0.8692

**Table 3:** DSC parameters of PL and MPL samples.

Samples	*T*_o_/°C	*T*_p_/°C	*T*_c_/°C
MPL_30_	35.50	79.17	149.17
MPL_40_	34.33	95.00	155.50
MPL_50_	36.50	91.50	156.50
MPL_60_	36.00	93.33	159.67
MPL_70_	37.17	93.67	169.50
PL	35.17	82.17	152.67

Note: *T*_o_, onset temperature; *T*_p_, peak temperature; *T*_c_, completion temperature.

## Data Availability

The original contributions presented in this study are included in the article. Further inquiries can be directed to the corresponding author.

## Abbreviations

The following abbreviations are used in this manuscript:

CTDS	Colon-targeting delivery system
FBG	Fasting blood glucose
FTIR	Fourier transform infrared
GI	Gastrointestinal
HDL-C	High-density lipoprotein cholesterol
LDL-C	Low-density lipoprotein cholesterol
LEfSe	Linear discriminant analysis effect size
MPLs	Microwave *Pueraria lobata* samples
OGTT	Oral glucose tolerance test
SCFA	Short-chain fatty acid
SEM	Scanning electron microscopy
SGF	Simulated gastric fluid
SIF	Simulated intestinal fluid
T2DM	Type 2 diabetes mellitus
TC	Total cholesterol
TG	Triglyceride
